# Modulation of circulating protein biomarkers following TRC105 (anti-endoglin antibody) treatment in patients with advanced cancer

**DOI:** 10.1002/cam4.207

**Published:** 2014-02-14

**Authors:** Yingmiao Liu, Mark D Starr, John C Brady, Andrew Dellinger, Herbert Pang, Bonne Adams, Charles P Theuer, Nam Y Lee, Herbert I Hurwitz, Andrew B Nixon

**Affiliations:** 1 Department of Medicine, Duke University Medical CenterDurham, North Carolina; 2 Department of Biostatistics and Bioinformatics, Duke University Medical CenterDurham, North Carolina; 3 School of Public Health, Li Ka Shing Faculty of Medicine, The University of Hong KongHong Kong, China; 4 Tracon Pharmaceuticals, Inc.San Diego, California; 5 Division of Pharmacology, College of Pharmacy, and Investigator, Davis Heart Lung Research Institute, Ohio State UniversityColumbus, Ohio

**Keywords:** Antiangiogenic therapy, biomarker, endoglin, phase I clinical trial, TRC105

## Abstract

TRC105 is an endoglin-targeting drug that possesses anti-angiogenic and antitumor potential. Analysis of the initial phase I trial of TRC105 demonstrated good tolerability and efficacy in cancer patients. In this report, we analyzed multiple circulating biomarkers at baseline, cycle 2 day 1 (C2D1), and end of study (EOS) for each patient. The baseline level and the fold change from baseline to both C2D1 and EOS for each marker were statistically analyzed. At C2D1, seven markers were significantly downregulated (angiopoietin-2 [Ang-2], insulin-like growth factor-binding protein-3 [IGFBP-3], plasminogen activator inhibitor-1 [PAI-1] total, platelet-derived growth factor [PDGF]-AA, PDGF-BB, thrombospondin-1 [TSP-1], and vascular endothelial growth factor [VEGF]-D). Meanwhile, seven markers were upregulated by C2D1 (E-Cadherin, soluble Endoglin [sEnd], E-Selectin, interleukin-6 [IL-6], osteopontin [OPN], TSP-2, and von Willebrand factor [vWF]). At EOS, seven markers were upregulated including Ang-2, C-reactive protein (CRP), intercellular adhesion molecule-1 (ICAM-1), IGFBP-1, IL-6, TSP-2, and vascular cell adhesion molecule-1 (VCAM-1). A statistical trend was also seen for increases of VEGF-A and placenta growth factor (PlGF) at EOS. Throughout treatment, sEnd levels significantly increased, an observation that was recapitulated in cultured endothelial cells. This is the first report of plasma-based biomarkers in patients receiving TRC105. TRC105 treatment by C2D1 was associated with decreases in several angiogenic factors, including Ang-2, PDGF isoforms, and VEGF isoforms, offering insight into the mechanisms underlying TRC105's anti-angiogenic, antitumor function. Increases in sEnd were the most significant of all observed biomarker changes and may reflect direct drug effects. Additionally, biomarker changes in response to TRC105 are distinct from those seen in patients treated with VEGF-targeting drugs, suggesting the possible utility of combining these two classes of angiogenesis inhibitors in patients.

## Introduction

Endoglin (CD105) is a membrane-bound glycoprotein highly overexpressed on proliferating endothelial cells 1. Endoglin is a standard marker used to identify proliferating vasculature, and localizes with ALK1 on cell membranes to phosphorylate Smad 1, 5, and 8 to facilitate endothelial cell proliferation and migration 2,3. In tumorigenesis, endothelial activation is indispensable for primary tumor growth and metastasis. Endoglin is densely expressed on the vasculature of more than 10 types of solid tumors, and its expression correlates with poor prognosis 4,5. Endoglin expression is upregulated in response to hypoxia induced by agents that inhibit vascular endothelial growth factor (VEGF) signaling 6,7, and tumors deprived of endoglin exhibit a delayed onset of resistance to anti-VEGF agents 8. Targeting endoglin, therefore, represents a novel approach to inhibiting angiogenesis and tumor growth, and complements the use of existing angiogenesis inhibitors that disrupt the VEGF pathway.

TRC105 is a monoclonal antibody that binds endoglin with high avidity and exhibits anti-angiogenic and antitumor effects in vitro and in vivo 9–11. A first-in-human, phase I trial demonstrated evidence of activity in patients with advanced solid tumors 12. Stable disease or better was achieved in 47% of patients, including two patients who achieved radiographic improvement for more than 18 months following treatment. Despite encouraging results in unselected patients, it is desirable to identify patients most likely to benefit from TRC105. In addition, the ability to detect markers of resistance at progression may enable clinicians to modulate treatment in a timely manner to improve clinical outcome. Blood-based biomarkers are one approach to address these challenges 13. Multiplex techniques allow the detection of multiple biomarkers, yielding a vast body of information regarding the drug targets, as well as crucial proteins involved in angiogenesis, inflammation, and extracellular matrix (ECM) remodeling.

Despite the recognized importance of biomarkers, the lack of consistency in approaching biomarker analyses continues to confound interpretation of results. There is a need for harmonization of scientific methodologies to better evaluate data across different trials, drugs, and patients. To facilitate this goal, our laboratory has been designated as a Molecular Reference Laboratory for the Alliance cooperative group (formerly CALGB). We have developed a versatile, multiplex panel that evaluates greater than 40 plasma-based biomarkers in cancer patients. This panel has been applied in a consistent manner to smaller, phase I/II studies as well as larger, randomized phase III studies.

In one of the largest studies to date, we utilized this approach in assessing the phase III trial of gemcitabine ± bevacizumab in metastatic pancreatic cancer (CALGB80303). We identified several factors that were highly prognostic for outcome in general, including insulin-like growth factor-binding protein-1 (IGFBP-1), intercellular adhesion molecule-1 (ICAM-1), angiopoietin-2 (Ang-2), C-reactive protein (CRP), interleukin-8 (IL-8), thrombospondin-2 (TSP-2), vascular cell adhesion molecule-1 (VCAM-1), plasminogen activator inhibitor-1 (PAI-1) active, IGF-1, and IL-6 14. Several analytes were found to be predictive of benefit or lack of benefit from bevacizumab, including VEGF-D, as well as stromal cell-derived factor-1 (SDF-1) and Ang-2. Importantly, the ability of VEGF-D to predict for benefit from bevacizumab was supported by the independent findings of the Australian GI Cancer Trials Group, who analyzed tissue VEGF-D by immunohistochemistry using archived formalin fixed, paraffin-embedded tumor samples 15. Recently, we completed a biomarker analysis of CALGB90206, a randomized phase III trial of interferon alfa-2B ± bevacizumab in patients with advanced renal carcinoma 16. In this study, we validated that hepatocyte growth factor (HGF) and IL-6 are predictive for bevacizumab, confirming results seen using pazopanib in renal cell carcinoma 17.

Here, we report biomarker data in 32 patients who received TRC105 in a phase I dose-escalation study 12. This is the first report of plasma-based biomarkers in patients treated with TRC105. We evaluated biomarker levels at baseline and early in treatment (4 weeks posttreatment with TRC105) to assess initial pharmacodynamic effects, as well as at progression (following discontinuation of TRC105) to assess potential mediators of resistance.

## Materials and Methods

### Patient grouping and drug formulation

Between January 2008 and February 2011, 50 patients were enrolled to receive TRC105 in this phase I, single arm trial. Written informed consent was obtained from each patient regarding the use of plasma for this correlative analysis. This study was IRB approved and registered with http://www.clinicaltrials.gov (study number: NCT00582985).

Based on the trial stage and shipping time, patient samples were divided into two groups. The first group consisted of 19 patients who received dosing levels of TRC105 between 0.01 to 3 mg/kg per 2 weeks. The drug TRC105 was initially produced in the mouse myeloma cell line NS0, which raised immunogenicity concerns as human anti-murine antibodies (HAMA) and human anti-chimeric antibodies (HACA) were detected in 9.5%, and 35% of patients, respectively. As such, TRC105 formulation was shifted to Chinese hamster ovary cells (CHO cells) and thereafter neither HAMA nor HACA were detected in patients. The second group of patients tested for biomarker profiling mainly received CHO-produced TRC105, ranging from 0.3 mg/kg per 2 weeks up to 15 mg/kg per week. This group consisted of 32 patients. The relevant clinical dosing of TRC105 was reached and the recommended doses for phase II analysis were found to be 10 mg/kg weekly and 15 mg/kg every 2 weeks. With the NS0-produced TRC105, the majority of patients received lower doses of drug; with the CHO-produced TRC105, most patients were treated at or near the recommended phase II dose.

Biomarker data from the first group (i.e., low dose group) has been presented previously 18. Here, we focus on biomarker data from the second group (i.e., high-dose group). Table 1 lists the patient characteristics for the high-dose group as well as the study as a whole.

**Table 1:** Characteristics of all cancer patients, and the subgroup of high-dose TRC105-treated patients whose biomarker data are reported

Characteristic	All patients (*n* = 50)	Patients in high-dose group (*n* = 32)
Age, median (range)	63 (25–83)	63 (25–83)
Gender		
Male	34 (68%)	24 (75%)
Female	16 (32%)	8 (25%)
Race		
Caucasian	38 (76%)	25 (78%)
African American	7 (14%)	1 (3%)
Others	5 (10%)	6 (19%)
ECOG at baseline		
0	15 (30%)	5 (16%)
1	35 (70%)	27 (84%)

### Plasma collection, handling, and storage

Blood was collected from each patient by venipuncture into either a sodium citrate vacutainer (BD Vacutainer, catalog # 369714; San Jose, CA), or an ethylene diamine tetra acetic acid (EDTA) vacutainer (BD Vacutainer, catalog # 367899), and mixed thoroughly. After mixing, the tubes were centrifuged at 2500*g* for 15 min. The upper layer of plasma was transferred to a fresh tube and centrifuged one more time at 2500*g* for 15 min. The double-spun, platelet-poor plasma was aliquoted, snap frozen, and stored at −80°C at Fisher BioServices (Franklin, MA), and then shipped to the Duke Molecular Reference Laboratory (Durham, NC). Samples were further aliquoted based on specific assay requirements and stored at −80°C until use.

### Multiplex and enzyme-linked immunosorbent assay

All biomarkers were measured using the SearchLight multiplex platform (Aushon Biosystems, Inc., Billerica, MA) (40 analytes, Table 2), except for transforming growth factor (TGF)-*β* R3 (R&D Systems, Inc., Minneapolis, MN), as reported previously 19.

**Table 2:** Levels of biomarkers at baseline, C2D1, and EOS

		Baseline		C2D1			EOS		
				
Biomarker	Unit	Median	Range	Median	Range	FC	Median	Range	FC
Ang-2	pg/mL	546.0	215.0–1072.1	451.8	204.4–1130.3	0.9	598.4	141.9–1350.8	1.3
CRP	*μ*g/mL	5.3	0.3–199.8	5.3	0.2–64.1	1.5	14.4	0.5–181.6	8.1
D-dimer	*μ*g/mL	34.6	15.8–102.6	33.5	21.1–72.5	1.1	36.4	20.0–65.4	1.2
E-Cadherin	ng/mL	42.0	17.0–123.5	48.4	24.0–142.9	1.3	55.2	16.4–134.6	1.5
E-Selectin	ng/mL	37.9	14.8–149.0	40.5	19.4–176.7	1.3	47.6	22.3–93.0	1.6
Endoglin	ng/mL	21.0	14.4–33.2	39.2	18.8–115.5	2.7	34.4	17.1–124.0	2.3
GRO-*α*	pg/mL	46.0	11.2–18856.0	36.8	11.0–18856.0	1.0	51.1	16.1–9090.6	1.2
HGF	pg/mL	170.3	85.2–13348.3	161.0	59.8–13124.1	1.1	187.3	58.4–12864.1	3.0
ICAM-1	ng/mL	357.1	246.4–716.4	357.9	233.7–835.8	1.0	423.4	253.4–1070.7	1.2
IGFBP-1	ng/mL	6.3	0.3–75.8	6.4	0.1–32.9	1.2	10.1	0.5–51.3	4.6
IGFBP-2	ng/mL	837.0	328.8–71383.9	998.4	322.4–71383.9	2.2	824.0	344.3–71383.9	1.0
IGFBP-3	ng/ml	488.9	191.8–786.7	407.9	113.2–807.3	0.9	442.3	45.6–853.0	0.9
IL-6	pg/mL	6.3	1.2–2072.1	8.6	1.4–1116.6	1.5	59.8	1.5–602.1	7.0
MCP-1	pg/mL	283.6	96.5–652.6	301.8	117.9–706.1	1.1	495.1	132.0–732.0	1.5
MMP-2	ng/mL	110.9	12.8–191.2	118.9	77.7–205.2	1.5	125.9	61.4–189.0	1.1
MMP-9	ng/mL	81.3	21.9–1031.1	97.7	28.1–737.4	1.3	65.8	24.1–213.0	1.0
OPN	ng/mL	101.8	41.9–414.6	123.9	34.1–329.0	1.1	111.9	38.9–275.7	1.3
PAI-1 active	ng/mL	3.2	1.1–123.0	2.3	0.8–123.0	1.2	3.2	1.0–111.9	2.6
PAI-1 total	ng/mL	19.9	5.0–74.9	16.3	4.5–40.1	0.9	18.1	6.8–57.5	1.2
PDGF-AA	pg/mL	139.4	4.7–2544.5	77.1	6.6–1955.5	0.9	101.3	7.3–707.8	1.7
PDGF-BB	pg/mL	287.8	27.6–1354.3	227.6	20.5–1182.4	1.0	302.6	39.8–1854.8	1.9
PEDF	*μ*g/mL	7.0	2.8–8.9	7.0	2.7–7.9	1.0	6.8	2.6–8.7	1.0
PIGF	pg/mL	7.4	0.8–1566.5	6.1	3.3–702.0	1.0	7.6	4.3–441.1	1.1
P-Selectin	ng/mL	117.4	54.1–1290.4	110.9	46.4–1290.4	1.1	122.2	63.3–952.1	1.3
SDF-1	pg/mL	589.9	14.6–12220.5	575.8	20.2–4362.1	1.3	588.0	106.6–1716.3	1.9
TF	pg/mL	34.4	4.7–27330.5	34.0	4.7–14285.6	1.4	46.6	2.2–5670.0	1.1
TGF-*β*1	ng/mL	28.1	4.3–92.7	23.8	8.3–73.5	1.1	28.6	4.8–45.5	1.4
TGF-*β*2	pg/mL	29.8	3.1–2848.5	28.4	4.7–2471.8	1.0	28.4	3.1–1958.7	1.3
TGF*β*-R3	ng/mL	64.0	32.5–113.9	68.5	39.2–211.0	1.1	78.3	44.3–167.0	1.2
TSP-1	ng/mL	53.9	7.4–551.4	48.7	4.4–393.6	0.8	51.8	9.4–302.2	1.3
TSP-2	ng/mL	18.5	5.7–146.6	18.5	5.4–82.0	1.2	26.4	9.8–127.7	1.6
VCAM-1	*μ*g/mL	2.0	0.9–4.7	2.0	1.2–5.9	1.1	2.6	1.1–5.4	1.4
VEGF-A	pg/mL	49.5	15.8–9192.2	64.2	3.8–5099.4	1.2	107.0	9.6–5701.2	1.8
VEGF-D	pg/mL	531.7	81.3–71697.5	415.1	17.1–53197.5	0.9	704.9	282.4–26746.8	1.6
sVEGF-R1	pg/mL	38.4	4.8–96186.3	32.8	3.0–42506.8	1.5	43.9	3.0–15428.2	2.4
sVEGF-R2	ng/mL	6.6	2.5–269.2	6.3	2.7–203.4	1.0	6.9	4.2–116.1	1.1
vWF	U/mL	8.4	3.3–201.3	8.7	3.1–227.5	1.4	13.1	4.4–47.1	1.6

Median levels and ranges for each marker are shown at all time points. Fold change (FC) from baseline is calculated for each individual patient at C2D1 and EOS, and the averaged FC across all patients is presented. Ang-2, angiopoietin-2; CRP, C-reactive protein; GRO-*α*, growth-related oncogene-alpha; HGF, hepatocyte growth factor; ICAM-1, intercellular adhesion molecule-1; IGFBP, insulin-like growth factor-binding protein; IL-6, interleukin-6; MCP-1, monocyte chemotactic protein-1; MMP, matrix metallopeptidase; OPN, osteopontin; PAI-1, plasminogen activator inhibitor-1; PDGF, platelet-derived growth factor; PEDF, pigment epithelium-derived factor; PlGF, placenta growth factor; SDF-1, stromal cell-derived factor-1; TF, tissue factor; TGF, transforming growth factor; TSP, thrombospondin; VCAM-1, vascular cell adhesion molecule-1; VEGF, vascular endothelial growth factor; sVEGF-R, soluble VEGF receptor; vWF, von Willebrand factor.

### sEnd assay

Initially, TRC105 was assessed for potential interference in both the R&D Quantikine CD105 Immunoassay kit (Catalog # DNDG00) as well as the Aushon CD105 Searchlight Immunoassay kit. Healthy volunteer plasma was titrated with increasing amount of TRC105 and tested in both kits following manufacturer's protocols. In the R&D assay, measured sEnd levels were decreased by 20% compared to no-TRC105 controls when the molar ratio of sEnd:TRC105 reached 1:100. Higher molar excesses of TRC105 (estimated molar ratio of sEnd:TRC105 ≥ 1:1000) completely abolished sEnd detection in the R&D assay format. In contrast, sEnd detection was not appreciably affected using the Searchlight assay, even when the molar ratio of sEnd:TRC105 reached 1:10,000 (data not shown). As such, the Searchlight assay was utilized to detect sEnd levels.

### Cell culture

Low passage human umbilical vein endothelial cells (HUVECs) from Clonetics/Lonza (Walkersville, MD) were cultured in endothelial basal medium supplemented with Quot Kit supplements and growth factors. HUVEC were inoculated onto a 12-well plate at about 50% confluence, and treated with TRC105 for 2 days. Then cell supernatants were collected, centrifuged once to remove cellular debris and stored at −80°C. HUVEC cell lysates were harvested in lysis buffer (20 mmol/L Hepes, 2 mmol/L MgCl_2_, 1 mmol/L EDTA and ethylene glycol tetra acetic acid, 150 mmol/L NaCl, 1% Triton X-100, 0.1% SDS [sodiumdodecyl sulfate], protease, and phosphatase inhibitors), centrifuged twice at 20,000 *g* for 10 min, and protein concentration determined (Bradford protein assay, Bio-Rad Life Science, Hercules, CA). A twofold dilution of supernatants and 1 *μ*g cell lysate per well were assessed using the SearchLight sEnd assay.

### RT-PCR

HUVEC cells were treated with TRC105 for 2 days, harvested, and washed once with cold phosphate buffered saline. RNA was extracted with a TaqMan Gene Expression Cells-to CT kit (Ambion, Carlsbad, CA). RT-PCR (polymerase chain reaction) was performed following manufacturer's protocol, with primers specific for Endoglin (Life technologies, Grand Island, NY).

### Statistical analysis

Analyses were performed on all patients for whom the relevant data were available. To evaluate on-treatment changes, L-ratio was calculated using the formula Log_2_ (post-treatment level/baseline level) for each analyte. Waterfall plots were produced for L-ratio to demonstrate analyte changes between the time points for all 32 patients. Signed-rank tests were used to determine statistical significance of biomarker changes, where *P* < 0.05 indicated significance; and 0.05 < *P* < 0.15 indicated a strong trend. Spearman correlations were calculated for all pairs of analytes at both baseline and L-ratio. Hierarchical clustering approaches were used to group the analytes into the provided dendrograms.

## Results

### Significant changes in biomarker levels in response to TRC105

Fifty patients with various advanced solid tumor types were enrolled in a phase I dose-escalation trial of TRC105. The data presented here focus on the plasma biomarker data from 32 patients who received doses of TRC105 from 0.3 to 15 mg/kg every 2 weeks as well as some patients receiving 10 and 15 mg/kg weekly. Results from the earlier cohort of 19 patients receiving lower doses of TRC105 (0.01–3 mg/kg per 2 weeks) have been presented previously 18. Overall, the patterns of change across the biomarkers analyzed are consistent between the two groups. Compared to the study as a whole, the patients reported here exhibited no apparent differences with regard to age, gender, race, and Eastern Cooperative Oncology Group (ECOG) performance status (Table 1).

In total, 41 biomarkers for each patient were evaluated at baseline, cycle 2 day 1 (C2D1), and end of study (EOS). Four markers (bone morphogenetic protein [BMP]-9, fibroblast growth factor basic, IL-8, and VEGF-C) were excluded from statistical analysis because more than 10% of samples were below the limit of detection. The median levels, ranges, fold changes from baseline for each of the 37 biomarkers are shown in Table 2. Assays for most markers evaluated were highly reproducible with coefficients of variation (CVs) in the 5–20% range (data not shown).

**Figure 1:**
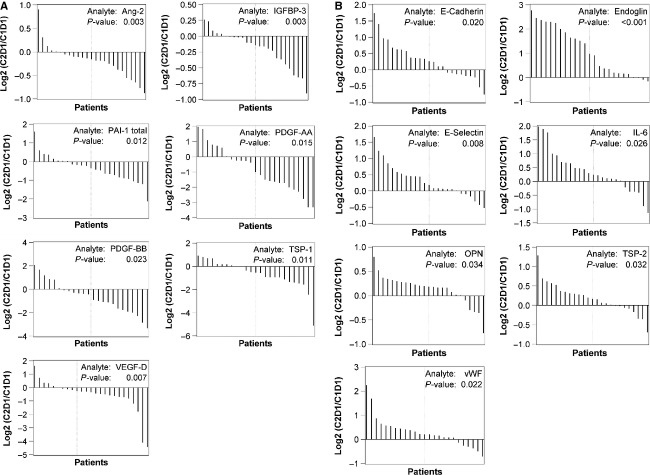
Waterfall analyses of changes from baseline to C2D1 for biomarkers with statistical significance (*P* < 0.05). (A) Downregulated biomarkers at C2D1. (B) Upregulated biomarkers at C2D1.

Biomarker levels at baseline and at C2D1 were compared to assess treatment-related changes. Seven biomarkers were significantly decreased at C2D1 compared to baseline, including Ang-2, IGFBP-3, PAI-1 total, platelet-derived growth factor (PDGF)-AA, PDGF-BB, TSP-1, and VEGF-D (*P* < 0.05) (Fig. 1A). Seven markers were significantly increased, including E-Cadherin, sEnd, E-Selectin, IL-6, osteopontin (OPN), TSP-2, and von Willebrand factor (vWF) (*P* < 0.05). Among the increased markers, the elevation of sEnd was the most robust. Twenty-one of 25 patients experienced up to eightfold increases in sEnd levels, while the remaining four patients exhibited slight reductions (Fig. 1B).

**Figure 2:**
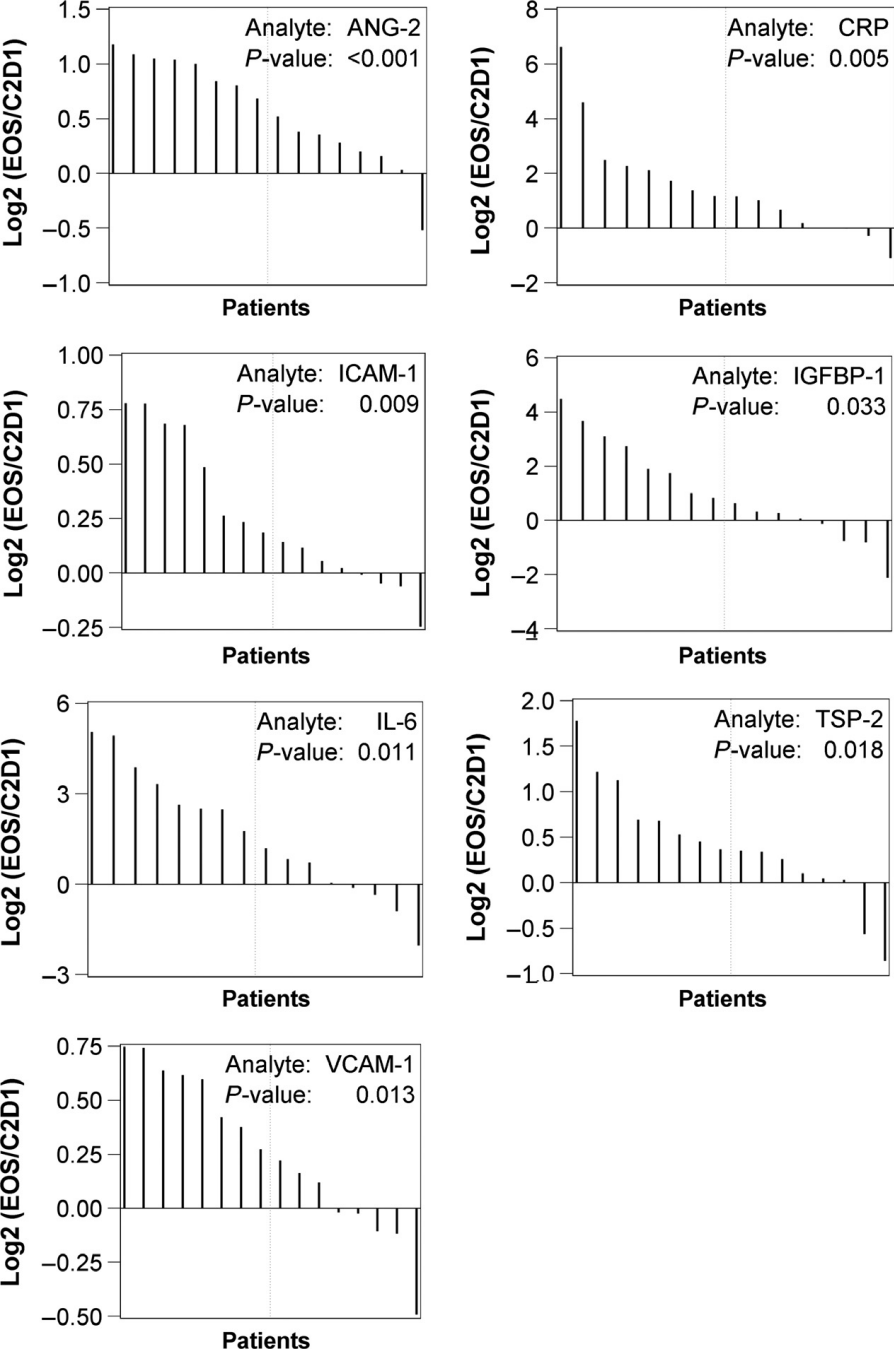
Waterfall analyses of change from C2D1 to end of study (EOS) for biomarkers with statistical significance (*P* < 0.05).

At EOS, the majority of markers were elevated when compared to C2D1. Statistically significant increases (*P* < 0.05) were detected in Ang-2, CRP, ICAM-1, IGFBP-1, IL-6, TSP-2, and VCAM-1 (Fig. 2). Note that for Ang-2, the decrease between baseline and C2D1, as well as the increase between C2D1 and EOS were both statistically significant (*P* < 0.05). Additionally, continuous increases were observed for IL-6 and TSP-2 from baseline to C2D1, and from C2D1 to EOS.

### Changes in VEGF family members in response to TRC105

VEGF family members are the most extensively studied markers in regards to anti-angiogenic therapies. In this trial, VEGF-A was decreased at C2D1 in response to TRC105 and subsequently increased at EOS (*P* = 0.101 and 0.144, respectively). It should be noted that the fold change (Table 2) reflects the average change across all patients. Therefore, a fold change >1 does not necessarily mean an overall increase. In the case of VEGF-A, 17 of 24 patients showed a reduction at C2D1, while 10 of 16 patients showed an increase at EOS. Although these changes did not reach statistical significance, they represent a strong trend. The related family member, VEGF-D, was also decreased at C2D1 (*P* = 0.007). Placenta growth factor (PlGF), another growth factor in the VEGF family, was decreased at C2D1 (*P* = 0.085), and increased at EOS (*P* = 0.058). The same pattern of change was seen across all three VEGF-related factors mentioned above: initial reduction at C2D1 followed by an elevation at EOS.

Soluble VEGF-R1 showed a trend for decreasing at C2D1 in 16 of 25 patients (*P* = 0.114). No apparent changes were detected in soluble VEGF-R2 at C2D1, but 11 of 16 patients exhibited a trend for increase between C2D1 and EOS (*P* = 0.117). Taken together, these data indicate a potential dampening of VEGF signaling pathways in response to TRC105 treatment. These data also demonstrate elevations in multiple VEGF ligands at the time of resistance to TRC105.

### Correlation among biomarkers

**Figure 3:**
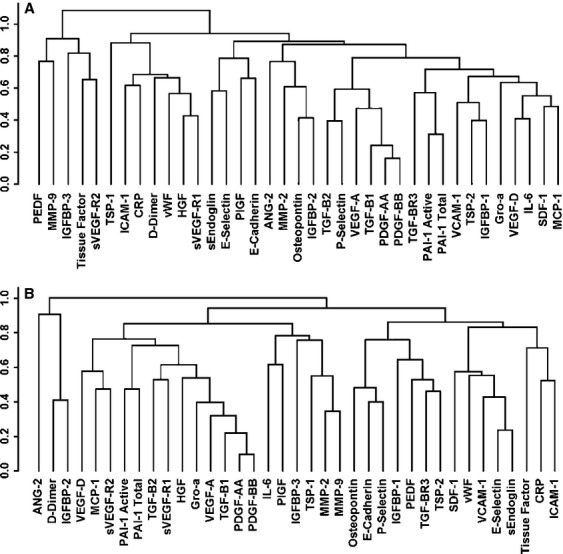
Dendrogram plots demonstrate hierarchical clustering patterns of biomarkers. (A) Baseline. (B) On-treatment.

Spearman's rank-order analyses were used to test pairwise correlations among the measured proteins in an attempt to better understand the potential coregulation of these specific biomarkers. At baseline, statistically significant pairs of markers (correlation coefficients ≥0.75, *P* < 0.001) included PDGF-AA and PDGF-BB, TGF-*β*1 and PDGF-AA, TGF-*β*1 and PDGF-BB. After one cycle of treatment, significant correlations were identified in following pairs: PDGF-AA and PDGF-BB, TGF-*β*1 and PDGF-AA, VEGF-A and PDGF-BB, Endoglin and E-Selectin. These correlations were all positive, indicating that paired biomarkers levels were either both high (or increased) or both low (or decreased). The correlations across all biomarkers at baseline, as well as on-treatment, are graphically illustrated in the dendrogram plots shown in Figure 3.

### Dose-dependent increase in sEnd in patients receiving high-dose TRC105

**Figure 4:**
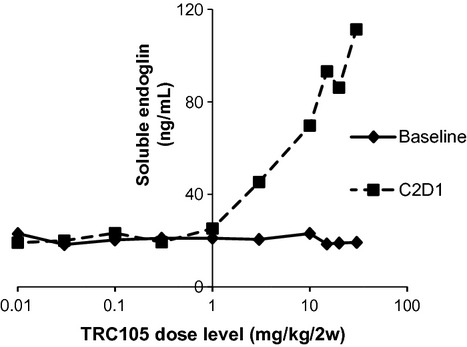
Soluble endoglin (sEnd) levels in patients' plasma increased in a TRC105 dose-dependent manner.

The effect of TRC105 on sEnd release is of great interest due to the fact that TRC105 targets membrane-bound endoglin. We demonstrated that in the SearchLight system, TRC105 will not affect the quantification of sEnd (see Material and Methods). Using this assay, we observed that sEnd levels did not fluctuate much across the low-dose group patients (TRC105 dosing below 3 mg/kg per 2 weeks) (Fig. 4). For the 32 patients in high-dose group, baseline levels of sEnd were comparable to those observed in low-dose group, at ∼20 ng/mL. However, in sharp contrast, when TRC105 was administrated at doses ≥3 mg/kg per 2 weeks, sEnd levels at C2D1 increased in a TRC105-dose-dependent manner (Fig. 4). sEnd increases persisted through C2D22 and EOS (data not shown), and were found to be highly significant (*P* < 0.0001; *r*-squared = 0.84).

### TRC105 induces sEnd level in HUVEC cell supernatant

**Figure 5:**
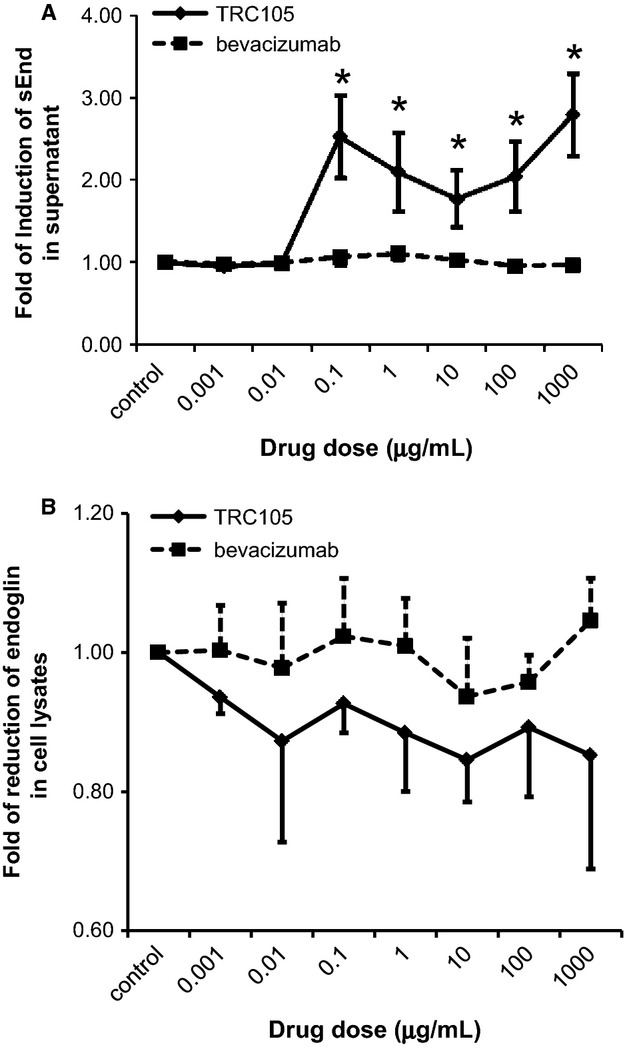
sEnd increased in response to TRC105 treatment in HUVEC cells. (A) sEnd induction in supernatant in response to 2 days treatment of TRC105, but not to the control, bevacizumab. (B) Cell-associated endoglin levels in lysates. Quantified data normalized to no drug control from three independent experiments ±SD are presented. *P < 0.05. sEnd, soluble endoglin; HUVEC, human umbilical vein endothelial cells.

Given our findings that TRC105 elevates sEnd in patient plasma, we next wanted to determine whether TRC105 leads to sEnd release in vitro using HUVEC as a model system. As shown in Figure 5A, low-dose TRC105 (0.001–0.01 *μ*g/mL) had no effect on sEnd after 48 h of treatment. However, 0.1 *μ*g/mL TRC105 significantly induced a threefold increase in sEnd in HUVEC supernatant (*P* < 0.05). Interestingly, at TRC105 doses of 1, 10, 100 *μ*g/mL, sEnd levels in the supernatant were less than what was observed for 0.1 *μ*g/mL. When TRC105 dose was further increased (1000 *μ*g/mL), sEnd levels again were increased threefold, as observed with 0.1 *μ*g/mL of TRC105. As a control, bevacizumab was tested over the same range of concentrations and exhibited no induction of sEnd. Correspondingly, levels of cellular endoglin were reduced by 10–20% in HUVEC cell lysates in response to TRC105, but not to bevacizumab (Fig. 5B).

Lastly, we investigated whether mRNA levels of endoglin were affected by TRC105 treatment. RT-PCR analysis revealed no apparent changes of endoglin RNA levels in response to TRC105 (data not shown), suggesting sEnd induction in cell supernatant was not due to enhanced transcription.

## Discussion

TRC105 is a novel anti-angiogenic antibody targeting endoglin and is currently being tested in phase Ib and phase II trials. The work presented here represents the first analysis of the effect(s) of TRC105 on plasma biomarkers in cancer patients. In this study, we focused our analysis on three time points: baseline, C2D1, and EOS. Baseline biomarkers levels reflect basal levels of circulating proteins and serve as each patient's own control. At C2D1, patients had received 4 weeks of TRC105 treatment and this point reflected the steady state of TRC105 12. EOS, on the contrary, occurred at different times for each patient (typically after 2 months of treatment), and was often marked by disease progression. Alterations in angiogenic factors assessed at this time point may likely reflect a condition of drug resistance.

Compared to baseline, seven angiogenic markers were significantly decreased at C2D1, including Ang-2, PDGF-AA, PDGF-BB, and VEGF-D (Fig. 1A). This downregulation was consistently observed in both low- and high-dose groups, and was statistically significant (*P* < 0.05). All four factors play pivotal roles in angiogenesis. Ang-2 is a crucial factor promoting pathological neoangiogenesis 20. PDGF-AA and -BB mediate the recruitment of pericytes to vascular endothelial cells 21. VEGF-D is a strong angiogenic and lymphangiogenic inducer 22. All these factors have been reported to be overexpressed in various solid tumors 23–25. Reduction in these factors at C2D1 likely reflects a broad downregulation of multiple angiogenesis pathways, confirming the proposed antiangiogenic role of TRC105.

Our analysis also revealed a strong correlation of TGF-*β*1, PDGF, and VEGF-A (Fig. 3). PDGF family members mediate a variety of biological responses, including proliferation and chemotaxis of smooth muscle cells and fibroblasts 21. TGF-*β*1, in the context of tumorigenesis, is a strong inducer of tumor cells proliferation, angiogenesis, and metastasis 26. TGF-*β*1 exhibited a strong downregulation trend in the majority of patients treated with all doses of TRC105 (*P* = 0.064 and 0.149 in the low and high-dose group, respectively). As PDGFs and VEGF-A have been shown to be regulated by TGF-*β*1 27, the concurrent reduction in TGF-*β*1, PDGF-AA and PDGF-BB, and VEGF-A suggests an important interplay among these factors in cancer patients treated with TRC105. The mechanism and impact of such interaction awaits further investigation.

We observed that compared to baseline, seven factors were significantly increased at C2D1: E-Cadherin, sEnd, E-Selectin, IL-6, OPN, TSP-2, and vWF (Fig. 1B). Interestingly, except for IL-6, a well-known inflammatory marker, the other factors could be grouped as matricellular proteins of the ECM 28,29. Characteristically, matricellular proteins do not form actual ECM structures (such as collagens, laminins and fibronectin), yet they have multifaceted regulatory roles, including the modulation of cell–cell and cell–matrix interactions. The impact of the overall induction of these matricellular factors is currently unknown, yet it is intriguing given that endoglin itself possess an arginine-glycine-aspartate motif at its extracellular domain, thereby having the potential to bind integrins and to act as an adhesion molecule 30. In addition, Tian et al. reported that endoglin is a mediator of the crosstalk between fibronectin/*α*5*β*1 integrin and TGF-*β*1 signaling pathways 31.

Among all upregulated biomarkers, the induction of sEnd was observed to be the most robust. sEnd increased fourfold to fivefold after treatment at the recommended phase II doses of TRC105 (i.e., 15 and 20 mg/kg per 2 weeks), reaching plasma concentrations of 0.1 *μ*g/mL (Fig. 4). Plasma levels of TRC105 were between 10 and 500 *μ*g/mL based on pharmacokinetic analysis 12. Roughly, the ratio of sEnd:TRC105 varies from 1:100 to 1:5000. Our titration assay indicated that TRC105 does not interfere with sEnd detection within this range. The marked increase in sEnd may be due to several factors, including prolonged stabilization of sEnd due to TRC105 binding or increased shedding of sEnd induced directly or indirectly by TRC105 binding at the cell membrane. Interestingly, no changes in sEnd levels have been noted in analysis of patient samples treated with other anti-angiogenic agents, including bevacizumab.

To further our understanding of TRC105-induced sEnd release, we recapitulated the release of sEnd in vitro using HUVEC cells. Soluble endoglin levels increased threefold in HUVEC cell supernatants after treatment with TRC105, but not to bevacizumab, confirming the induction of sEnd release is TRC105-specific. The induction appears to be biphasic, with the maximal induction accomplished at 0.1 *μ*g/mL (Fig. 5A). The target concentration of TRC105 for maximum effect in patients is 0.2 *μ*g/mL 12, very close to the dose that elicits significant induction of sEnd in our HUVEC experiments, suggesting that sEnd induction has potential implications for monitoring physiologically relevant target inhibition in patients. Moreover, recent reports show that sEnd can serve as scavenger or trap for circulating ligands, such as BMP-9 and -10 32, and can block downstream signaling pathways, thus impairing blood vessel sprouting and suppressing tumor growth 33.

Interestingly, we observed that higher doses of TRC105 (100–1000 *μ*g/mL) also exhibit strong induction of sEnd release. TRC105 exposure at this high-dose range effectively inhibits multiple HUVEC functional activities, including viability, migration, and tubular network formation (Y. Liu, H. Tian, G. C. Blobe, C. P. Theuer, H. I. Hurwitz, A. B. Nixon, unpubl. data). All of these mechanisms may contribute to the anti-angiogenic, antitumor function of TRC105.

Concurrent with sEnd release into the supernatant, a slight decrease (10–20%) of endoglin in HUVEC cell lysate was detected (Fig. 5B). RT-PCR analysis revealed no change in endoglin mRNA levels in response to TRC105 treatment, suggesting increased sEnd levels are not due to altered transcriptional activation. Rather, it could be that TRC105 binds to membrane-anchored endoglin, affects the rate of internalization, and triggers its release from cell surface. Matrix metallopeptidase (MMP)-14 has been identified as the main protease responsible for endoglin cleavage 34. It has been shown that TRC105 not only upregulates MMP-14 expression, also it facilitates colocalization of endoglin and MMP-14, leading to enhanced cleavage and accumulation of sEnd 35. Alternatively, the TRC105-sEnd complex may have a reduced clearance compared to free sEnd. Multiple mechanisms may be responsible for sEnd induction in patients' plasma as well as in HUVEC supernatant.

At EOS, seven markers demonstrated significant elevations compared with C2D1 levels, including previously decreased markers, such as Ang-2 (Fig. 2). Regarding the change in directions across the time points tested, two major patterns emerged. In some cases, biomarkers demonstrated a consistent increase with time. For example, IL-6 was upregulated at both C2D1 (*P* = 0.026) and EOS (*P* = 0.011). In other cases, markers decreased at C2D1 and then increased at EOS. For example, Ang-2 was reduced by C2D1 (*P* = 0.003) and induced at EOS (*P* < 0.001). The same pattern was also observed for PDGF-AA, PDGF-BB, and VEGF-D. These three markers exhibited significant downregulation at C2D1 (*P* = 0.015, 0.023, 0.007, respectively). Yet by EOS, most patients showed elevations in PDGF-AA, PDGF-BB, and VEGF-D levels. Although the interpretation of biomarker changes at baseline/C2D1 and C2D1/EOS is not conclusive, these biomarker changes may reflect a transition from a transient drug-sensitive state (C2D1) to an eventual drug-resistant state (EOS).

Biomarker studies following bevacizumab administration have been reported by our group and others 14,19,36,16. Although comparing biomarker changes in response to bevacizumab and TRC105 was not the objective of this study, several observations merit discussion. PlGF, a VEGF family member, is invariably upregulated following bevacizumab treatment 37. This has been interpreted as a direct on-target effect of VEGF-pathway blockage. In contrast, PlGF is downregulated in response to TRC105 (*P* = 0.045 and 0.085 in the low- and high-dose cohorts, respectively). VEGF-D, another VEGF family member, has been reported to increase in response to bevacizumab. Additionally, VEGF-D may potentially predict for bevacizumab benefit, as shown both in blood 14, and in tissue 15. In contrast to the data in bevacizumab-treated patients, VEGF-D was significantly reduced following administration of TRC105 (*P* = 0.003 and 0.007 in the low- and high-dose cohorts, respectively). Soluble VEGF-R2, a pivotal receptor for VEGF signaling, often decreases in response to anti-VEGF treatment 38. In contrast, soluble VEGF-R2 was not reduced in response to TRC105 at C2D1. Collectively these data demonstrate that TRC105 induces unique changes not only in sEnd, but also in other angiogenic factors, many of which are known to be TGF-*β*1 regulated. The apparent difference in these changes compared to those seen with bevacizumab and other VEGF inhibitors suggest that many of these changes are drug- and target-specific. Combinations of anti-VEGF and anti-endoglin therapies may have complementary anti-angiogenic effects, thus supporting the ongoing efforts to combine such agents in the clinic (see TRC105 trials listed in http://www.clinicaltrials.gov).
